# The role of ischemic preconditioning in the expression of apoptosis-related genes in a rat model of intestinal ischemia-reperfusion injury^1^

**DOI:** 10.1590/s0102-865020190050000001

**Published:** 2019-06-03

**Authors:** Celina Teresa Castelo Branco Couto de Miranda, Djalma José Fagundes, Edinaldo de Miranda, Ricardo Santos Simões, Adriana Aparecida Ferraz Carbonel, Rinaldo Florencio-Silva, Murched Omar Taha

**Affiliations:** I PhD, Assistant Professor, Medical School, Universidade Estadual do Piauí (UESPI), Teresina-PI, Brazil. Acquisition of data, manuscript preparation and writing.; II PhD, Full Professor, Division of Surgical Techniques and Experimental Surgery, Department of Surgery, Universidade Federal de São Paulo (UNIFESP), Brazil. Manuscript preparation and writing.; III PhD, Department of Morphology and Genetics, UNIFESP, Sao Paulo-SP, Brazil. Manuscript preparation and writing.; IV Postdoctoral, Department of Morphology and Genetics, UNIFESP, Sao Paulo-SP, Brazil. Manuscript preparation and writing.; V PhD, Associate Professor, Surgical Technique and Experimental Surgery, UNIFESP, Sao Paulo-SP, Brazil. Manuscript preparation, final approval.

**Keywords:** Ischemic Preconditioning, Apoptosis, Mesenteric Ischemia, Reperfusion, Rats

## Abstract

**Purpose:**

To analyze the effects of ischemic preconditioning (IPC) in the expression of apoptosis-related genes in rat small intestine subjected to ischemia and reperfusion.

**Methods:**

Thirty anesthetized rats underwent laparotomy and were drive into five groups: control (CG); ischemia (IG); ischemia and reperfusion (IRG); IPC and ischemia (IG+IPC); IPC and ischemia and reperfusion (I/RG+IPC). Intestinal ischemia was performed by clamping the superior mesenteric artery for 60 minutes, whereas reperfusion lasted for 120 minutes. IPC was carried out by one cycle of 5 minutes of ischemia followed by 10 minutes of reperfusion prior to the prolonged 60-minutes-ischemia and 120-minutes-reperfusion. Thereafter, the rats were euthanized and samples of small intestine were processed for histology and gene expression.

**Results:**

Histology of myenteric plexus showed a higher presence of neurons presenting pyknotic nuclei and condensed chromatin in the IG and IRG. IG+IPC and I/RG+IPC groups exhibited neurons with preserved volume and nuclei, along with significant up-regulation of the anti-apoptotic protein Bcl2l1 and down-regulation of pro-apoptotic genes. Moreover, Bax/Bcl2 ratio was lower in the groups subjected to IPC, indicating a protective effect of IPC against apoptosis.

**Conclusion:**

Ischemic preconditioning protect rat small intestine against ischemia/reperfusion injury, reducing morphologic lesions and apoptosis.

## Introduction

During ischemia blood flow is interrupted, leading to the deprivation of oxygen supply and metabolites/substrates^1^ ^,^ ^2^. Ischemia also leads an impairment in the elimination of metabolic wastes from cells such as reactive oxygen species (ROS)^2^ ^,^ ^3^. Increased ROS may lead to lipid peroxidation, a process that compromises the integrity of cell membranes and organelles including mitochondria, which can be irreversible^3^. This scenario may be aggravated by the restoration of blood flow (reperfusion), as there is the release of cell debris from cells damaged during ischemia. These cell debris attract a high number of macrophages, which then initiate an inflammatory process and consequently increased ROS production by macrophages^4^. Thus, the damages caused by reperfusion are due in part to macrophage activation and subsequent increased ROS production. Moreover, this process may cause endothelial damages and subsequent release of proinflammatory cytokine^5^. 

Intestinal ischemia and reperfusion (I/R) play important roles in the pathogenesis of systemic inflammatory response syndrome (SIRS) and multiple organ dysfunction syndrome (MODS)^6^ ^,^ ^7^. This is because I/R is associated with increased intestinal permeability, which is one of the causal factors of bacteria translocation to other locations outside intestine^8^. In addition, it is known that I/R results in intestine cell apoptosis in part as the result of increased ROS and proinflammatory cytokine production^9^. Accordingly, apoptosis after ischemia and reperfusion has also been observed in other organs such as brain^10^, heart^11^, lung^12^, and kidney^13^. 

On the other hand, it has been reported that IPC could mitigate the deleterious effects of I/R^14^ ^,^ ^15^. However, the mechanisms by which IPC could alleviate the tissue-damaging effects of I/R is poorly understood. The understanding of the mechanisms behind IPC protection against I/R requires the knowledge about changes in gene expression triggered by I/R. Thus, in this study, within our line of research on procedures and drugs that may have a relevant role in reducing or abolishing the deleterious damages of intestinal ischemia and reperfusion, we aimed to investigate the gene expression profile of apoptosis-related proteins in intestine cells in a rat model of intestinal ischemia-reperfusion injury.

## Methods

The experimental protocol was approved by the Animal Research Ethics Committee of the Universidade Federal de São Paulo (CEUA - UNIFESP), protocol number 1815/08. 

Thirty adults male Wistar rats (*Rattus norvegicus albinus*), with body weight between 250 and 300g, were provided by the Center for the Development of Experimental Models for Biology and Medicine (CEDEME-UNIFESP). The animals were housed in cages under standard laboratory conditions (12:12 light/dark cycle with lights on at 07:00 h; constant temperature of 23 ± 2°C, and humidity of 55 ± 10%) during one week for acclimation. Standardized chow and water were provided *ad libitum* until 6 h prior to the surgical procedures. With the rats under anesthesia (80 mg.kg^-1^ ketamine and 10 mg.kg^-1^ xylazine, injected intramuscularly), a median laparotomy exposed the superior mesenteric vessels. The rats were then randomly assigned into 5 groups (n = 6), as follows: control group (CG); ischemia group (IG); ischemia and reperfusion group (IRG); IPC followed by ischemia group (IG+IPC) and IPC followed by ischemia and reperfusion group (I/RG+IPC). Intestinal ischemia was performed by clamping the superior mesenteric artery for 60 minutes, whereas reperfusion lasted for 120 minutes. IPC was carried out by one cycle of 5 minutes of ischemia followed by 10 minutes of reperfusion prior to the prolonged 60-minutes-ischemia and 120-minutes-reperfusion. Ischemia was confirmed by observing the pale appearance of the clamped intestine and the lack of arterial beating. All animal procedures were carried out as previously described^7^.

Afterwards, segments of the jejunum of all animals were collected and fixed in 10% neutral buffered formalin solution for histological analysis. Other segments of the jejunum were removed and gently washed in PBS; after wrapped in aluminum foil and snap-frozen in liquid nitrogen, the frozen samples were stored in ultralow freezer for gene expression analyzes. Then, the animals were euthanized by anesthetic overdose.

### Histological procedures and hematoxylin and eosin staining

 After fixation for 24h in 10% neutral buffered formalin solution, samples of the jejunum were dehydrated in ascending concentrations of ethanol, cleared in xylene and embedded in paraffin. Cross-sections (5 µm-thick) of the jejunum were collected onto slides and stained with Hematoxylin and Eosin (H.E) for morphological evaluation. 

### Real-time PCR

 PCR was carried out according to the manufacture instructions. Breafly, total RNA was extracted by using Trizol reagent. Then, 2 μg of RNA was used for reverse transcription into cDNA and Real-time PCR was run in a thermal cycler (model MX3000P, Applied Biosystems). The assays were performed in 96-well plates to detect the expression of 84 genes related to Endothelial Cell Biology, which contain 28 genes related to apoptosis (PARN-015Z, QIAGEN)^7^. The conditions for PCR were 95°C for 5 min, 40 amplification cycles of 95°C for 30 s, 58°C for 30 s, and extension at 72°C for 45 s. β-actin served as an internal reference and the expression of target genes was normalized to that of β-actin. Comparative Ct threshold method and the ΔΔCt were used for relative quantification. Expression gene data were evaluated in triplicate for each sample. The results of gene expression were presented as positive expression/up-regulation (+), or negative expression/down-regulation (-). The software stablished the results three times above (hyper expression) or three times below (hypo expression) of the limit allowed by the algorithm [2^ (- ΔΔ Ct)], as a biologically meaningful way.

### Statistical analysis

 Statistical tests were carried out by using the Graphpad prism 5 software. Data are reported as mean and standard deviation (Mean ± SD). The one-way analysis of variance (ANOVA) followed by the Tukey post hoc test were performed to evaluate differences among groups. A *p* value of ≤ 0.05 was considered statistically significant.

## Results

### Histological analysis

**Figure 1:**
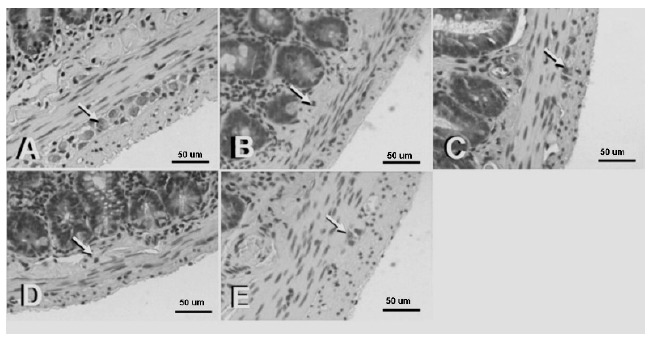
Photomicrographs of H.E stained histological sections from portions of the jejunum of rats from all groups: control (**A**), IG (**B**), I+IPC (**C**), I/RG (**D**) and I/R+IPC (**E**). A higher presence of neurons (*arrows*) with reduced volume and presenting pyknotic nuclei and condensed chromatin can be noticed in the IG (**B**) and I/RG (D) groups. Scale bar = 40 µm.

 The histological evaluation of the myenteric plexus showed higher presence of neurons presenting reduced volume, with pyknotic nuclei and condensed chromatin in the IG and IRG. Meanwhile, IG+IPC and IRG+IPC evidenced neurons with preserved volume and nuclei rich in euchromatin and evident nucleolus (Fig. 1).

### Real-time PCR

 Among the 28 apoptosis-related genes studied, only 10 were up-regulated or down-regulated (34.52%). The IG+IPC and IRG+IPC groups showed a significant up-regulation of the anti-apoptotic protein Bcl2l1, along with significant down-regulation of pro-apoptotic genes (Table 1). Moreover, Bax/Bcl2 ratio was lower in the groups subjected to IPC (Table 2). 

**Table 1:** Expression of genes related to apoptosis in the intestinal cells of rats: ischemic group (IG), ischemic and reperfusion group (I/RG), ischemic and IPC group (IG+IPC) and ischemic and reperfusion and IPC group (I/RG+IPC), compared to control group. Significant values of fold up (+) or down (-) regulation was highlighted in italicized [2^(-Delta Ct)].

Symbol	Gene	IG	IG+IPC	I/RG	I/RG+IPC
Bax	Protein Coding, BCL2 Associated X	-1.53	+2.30	-1.48	+5.45*
Bcl2	B-cell CLL/lymphoma	+14.06*	*+3.12*	*+3.86*	*+4.82*
Bcl2l1	Bcl2-like 1	-1.10	+3.48*	+1.01	+7.48**
Casp1	Caspase 1	+35,11*	+2.42	+100.68**	**+*3.85***
Casp3	Caspase 3, apoptosis related cysteine protease	+2.95	-1.87	+***8.28*** *	+1.35
Ccl5	Chemokine (C-C motif) ligand 5	+1.30	**-*5.44*** *	+2.70	**-*4.51*** *
Il1beta	Interleukin 1 beta	-1.95	**-*16.03*** *	+1.92	-8.59**
Il6	Interleukin 6	-8.09*	-20.08**	+3.52*	-14.74**
Cflar	CASP8 and FADD-like apoptosis regulator	+1.02	-5.55*	+41.60**	-4.68*
Faslg	Fas ligand (TNF superfamily, member 6)	+1.95	-4.63*	+2.40	-7.24**

Notes: *p<0.05; **p<0.001, for the sample in triplicate values.

**Table 2:** Relative gene expression of Bax/Bcl2L1 ratio related to intestinal cell biology from rats in the different groups.

	GROUPS			
Relation	IG	IG+IPC	I/RG	I/RG+IPC
Bax/Bcl2l1	1.39	0.66	1.45	0.73

## Discussion

Apoptosis plays important roles in regulating development, homeostasis and immune defense by removing abnormal cells from organisms. A balance between pro-apoptotic and anti-apoptotic mechanisms determines whether a cell can activate the apoptotic program.

Here we used the Rat Endothelial Cell Biology RT² Profiler PCR Array, which evaluate the expression of 84 genes related to endothelial cell biology. Among these 84 genes, 28 are directly related to apoptosis: Anxa5, Bax, Bcl2, Bcl2l1 (Bcl-xl), Casp1 (Ice), Casp3, Cav1, Ccl2 (MCP-1), Ccl5 (RANTES), Cflar (Casper), Cx3cl1, Edn1, Ednra, Fas (Tnfrsf6), Faslg (Tnfsf6), Fgf2 (bFGF), Hif1a, Il1b, Il3, Il6, Il7, Ocln, Pf4, Ptk2 (FAK), Tek (Tie-2), Thbs1 (TSP-1, Tsp1), Tnf, Tnfsf10 (Trail). Our results showed that among the 28 genes, only 10 (34.52%) were hyper expressed or hypo-expressed (Table 1).

There are an intricate cross-talk between the pro-apoptotic and anti-apoptotic mediators. For instance, Bax protein forms a heterodimer with Bcl2, which activates the apoptotic pathway. Bax increases the opening of the mitochondrial voltage-dependent anion channel (VDAC), leading to a loss in membrane potential and release of cytochrome “c” to the cytoplasm, thus activating the apoptotic cascade^16^. In our study, Bax was not down regulated in the groups subjected to IPC, suggesting that IPC stimulus is unable to interfere with Bax gene expression in our experimental conditions.

The Bcl-2 family of regulatory proteins of cell death has pro-apoptotic (Bax and Bak) and anti-apoptotic (Bcl2 and Bcl-XL) proteins^17^. In this study, Bcl-2 gene expression was up-regulated in all groups, but it was more evident in IG group, suggesting that Bcl-2 expression is not differentially stimulated by IPC. On the other hand, the Bcl2l1 protein also belongs to the Bcl-2 family and has the same action mechanism. It is a powerful inhibitor of apoptosis by in part inhibiting caspase activation. The expression of Bcl2l1 gene was significantly up-regulated three times above the threshold of normality. This suggests that the protective mechanism of IPC against apoptosis after I/R involves Bcl2l1, but not Bcl-2. 

According to Chueh *et al*.^18^ the protective effect of an agent against apoptosis can be assessed by observing the Bax/Bcl2 ratio. The lower its value, the lower is the chance of the cell to undergo apoptosis. In our study, the relation of Bax/Bcl2L1 gene expression in the IG+IPC and IRG+IPC groups was 0.66 and 0.73, respectively, indicating that IPC presented a protective effect against apoptosis.

The cystein-aspartic acid protease (caspase) family is involved in the signal transduction pathways of apoptosis, necrosis and inflammation^16^. There are two families of caspases: the inflammatory caspases and the apoptotic caspases, and each of these families can be further classified into initiator and effector subgroups. The initiator caspases (caspases 1, 4, 5, 8, 9, 10, 11 and 12), may be directly activated by death receptors such as Fas. Effector caspases (caspases 3, 6, and 7) are responsible for cleaving downstream substrates and are sometimes referred to as executioner caspases. Caspase 3 cleaves and activates caspases 6, 7 and 9, whereas caspase 3 itself is processed by caspases 8, 9 and 10^16^.

The first enzyme of caspase family described in mammals was ICE (interleukin-1b converting enzyme), currently known as caspase 1. The reason that lead to the identification of caspase 1 was its role in converting interleukin-1β, an important protein of the immune system. Thereafter, caspase 1 attracted more attention due to its role in apoptosis. The protein encoded by the IL1- β gene is a member of the interleukin 1 cytokine family. This cytokine is produced by activated macrophages as a proprotein, which is proteolytically converted into its active form by caspase 1 (CASP1/ICE). IL1- β is an important mediator of the inflammatory response and is involved in several cellular activities such as cell proliferation, differentiation, and apoptosis, as well as septic shock and wound healing^16^ ^,^ ^19^. 

In this study, a down regulation of IL1β and IL6 was seen in the groups subjected to IPC, which was related to less apoptotic activity. Accordingly, caspase 1 was up-regulated in the IG and IRG, and down-regulated in IPC groups. The reduction in caspase 1 expression in the IPC groups suggests that IPC could present protective effects against I/R injury, by inhibiting the activation of the caspase cascade. Moreover, we observed that caspase 3 gene expression was up-regulated in IRG, whereas it was down-regulated in the I/RG+IPC. Thus, these findings support the hypothesis that I/R-induced apoptosis is mitigated by IPC.

Ccl5-induced cell death involves the cytosolic release of cytochrome c, which activates caspase 9 and caspase 3, and poly (ADP-ribose) polymerase cleavage. Ccl5-induced apoptosis is CCR5-dependent, since native PM1 and MOLT4 cells lacking CCR5 expression are resistant to Ccl5-induced cell death ^20^. In this study, we observed hypo-expression of Ccl5 gene in IPC-subjected groups, suggesting that IPC could protect against Ccl5-induced apoptosis. 

The protein encoded by Cflar gene is a regulator of apoptosis and is structurally similar to caspase 8. However, the encoded protein lacks caspase activity and appears to be cleaved into two peptides by caspase 8. This apoptosis regulator protein is a crucial link between cell survival and cell death pathways in mammalian cells, whereby it acts as a regulator of TNFRSF6-mediated apoptosis^16^. We observed that the Cflar gene was down regulated in IG+IPC and IRG+IPC groups, while it was up regulated in IRG, indicating that the protective effect of IPC could be related to caspase 8 activity.

The Faslg is a member of TNF superfamily that binds to Fas, a cell death receptor located on the cell surface, thereby triggering the extrinsic apoptotic pathway^21^. The Fas/Faslg signaling pathway is essential in the regulation of the immune system, including the cell death activation of cytotoxic T cells^22^. In this study, the significant hypo-expression of Faslg gene in IPC groups evidences its protective effects by reducing inflammatory lesions and extrinsic apoptosis. 

We also performed a histological analysis of the neurons in the myenteric plexus, located between the muscular layers (inner circular layer and outer longitudinal layer) of the jejunum. We chose these cells due to their resistance to apoptosis. Neurons exhibiting morphological characteristics of apoptosis, such as reduced cell volume, pyknotic nuclei and condensed chromatin^23^ were frequently observed in the IG and I/RG groups. Meanwhile, the IPC groups exhibited neurons with preserved volume and nuclei rich in euchromatin and evident nucleoli. Thus, our histological findings also support the hypothesis that IPC could protect against I/R-induced-apoptosis. 

Taken together our findings indicate that IPC could alleviate the deleterious effects of the intestinal I/R, in part, by its ability to modulate the expression of apoptosis-related proteins. These effects involve the up-regulation of antiapoptotic genes and down-regulation of genes encoding proapoptotic proteins. 

## Conclusion

Ischemic preconditioning protects endothelial cell of rat intestine against ischemia/reperfusion injury, reducing morphologic lesions and apoptosis.
